# Defense Systems and Prophage Detection in *Streptococcus mutans* Strains

**DOI:** 10.1111/omi.70014

**Published:** 2025-11-11

**Authors:** Olivier Claisse, Cas Mosterd, Claire Le Marrec, Johan Samot

**Affiliations:** ^1^ University of Bordeaux, INRAE, Bordeaux INP, Bordeaux Sciences Agro, UMR 1366, OENO, ISVV Villenave d'Ornon France; ^2^ Department of Oral Surgery CHU Bordeaux Bordeaux France

**Keywords:** bioinformatics, immunology, *Streptococcus mutans*

## Abstract

Although the species is extensively studied, limited data are available on antiphage defense systems (APDSs) in *Streptococcus mutans*. The present study aimed to explore the diversity and the occurrence of APDSs and to search for prophages in the genomes of clinical isolates of *S. mutans* using bioinformatics tools.

Forty‐four clinical isolates of *S. mutans* were obtained from saliva samples of people with Parkinson's disease. Genomic DNA was extracted, sequenced using Illumina MiSeq technology, and analyzed for the presence of defense systems using DefenseFinder and PADLOC. CRISPR‐Cas systems were characterized using CRISPRCasFinder, and prophages were detected by the PhiSpy pipeline from RAST. AcrFinder and AcrHub were used to identify anti‐CRISPR proteins.

Each strain harbored between 6 and 12 APDS, with restriction‐modification systems being the most prevalent, followed by the MazEF toxin–antitoxin system and CRISPR‐Cas systems. Type II‐C CRISPR‐Cas systems were not identified here in *S. mutans*. Novel variations in type II‐A signature protein Cas9 were identified, allowing their classification into four distinct groups. Variability in direct repeat sequences within the same CRISPR array was also observed, and 80% of the spacers were classified as targeting “dark matter”. A unique prophage, phi_37bPJ2, was detected, showing high similarity with previously described phages. The AcrIIA5 protein encoded by phi_37bPJ2 was conserved and suggested to remain functionally active.

This study reveals the diversity of APDSs in *S. mutans* and the limited presence of prophages. The findings provide a foundation for future research on the evolutionary dynamics of these systems and their role in *S. mutans* adaptation to phage pressure.

## 1 Introduction

*Streptococcus mutans*, a pathogen commonly associated with caries, is one of the most extensively studied Gram positive bacteria. A bibliographic search on the PubMed database in early December 2024 found over 14,800 references. However, few bacteriophages are described for this bacterium in literature. Among the bacteriophages that have been described, lytic phages predominate including e10 and f1 (for which no complete sequence is available) (Delisle and Rostkowski 1993), M102 (van der Ploeg 2007), M102AD (Delisle et al. 2012), ΦAPCM01 (Dalmasso et al. 2015), and SMHBZ8 (Ben‐Zaken et al. 2021). Prophages have been described in genomes (Fu et al. 2017; Higuchi et al. 1982) but only one, ΦKSM96, has been isolated and sequenced (Sugai et al. 2023).

This relative scarcity of isolates is particularly striking given that metagenomic studies consistently reveal an abundance of viral sequences, including many phage genomes, in oral microbiomes, suggesting that the oral cavity could logically represent a hotspot for phage infection (Al‐Jarbou 2012; Jie et al. 2025). Several explanations may account for the difficulty of phage isolation in general, and for *S. mutans* phages in particular, ranging from methodological factors to host‐related barriers. On the bacterial side, one example in *S. mutans* is the rhamnose‐glucose polysaccharide (RGP), a major cell wall component that determines serotype and can interfere with phage adsorption (Shibata et al. 2009). In addition, *S. mutans* harbors more specific antiphage defense mechanisms. Although studies remain limited, CRISPR‐Cas is the best‐characterized system in this species, with subtypes II‐A, II‐C, I‐C, and I‐E reported (Lemaire et al. 2022). Over the past decade, however, numerous additional antiphage defense systems (APDSs) have been discovered across bacteria, greatly expanding the spectrum of known mechanisms. These systems act through highly diverse strategies, ranging from DNA degradation (e.g., Gabija, Hachiman) to translation inhibition (e.g., RloC, PrrC), or toxin–antitoxin‐like activities (e.g., RosmerTA) (Leprince et al. 2025; Millman et al. 2022).

Despite the many articles discussing *S. mutans*, little attention has been paid to the APDSs of this bacterium. More data on its defense systems could help understand how this bacterium copes with the pressure exerted by bacteriophages in its environment.

In this study, we analyzed the APDSs of 44 new *S. mutans* clinical oral isolates. We also report the presence of a complete prophage whose sequence was compared with those of other *S. mutans* phages.

## 2 Materials and Methods

### 2.1 Strains: Collection and Identification

Clinical isolates of *S. mutans* were isolated from saliva samples of people with Parkinson's disease. Participants were recruited in the PARKIDENT clinical trial (ClinicalTrials.gov Identifier: NCT03827551, https://clinicaltrials.gov/study/NCT03827551), whose research protocol was approved by a French regional ethics committee, the Comité de Protection des Personnes (CPP) Sud‐Est III (approval number EudraCT N° 2018‐A02773‐52) and for which all participants signed a written informed consent form.

Strains were identified as previously described (Donnet et al. 2025; Ziane‐Casenave et al. 2023). Briefly, saliva samples were diluted and plated on Mitis Salivarius Agar medium supplemented with potassium tellurite, bacitracin, and kanamycin (MSKB) to isolate bacteria. MSKB is selective for *S. mutans* due to its resistance to bacitracin and kanamycin, and its ability to reduce potassium tellurite, which results in the formation of distinctive gray to black colonies. Colonies were restreaked and characterized by Gram staining.

Presumptive *S. mutans* colonies were subsequently confirmed by MALDI‐TOF mass spectrometry using a Bruker MALDI Biotyper. Samples were prepared using the direct transfer‐formic acid method. Briefly, a portion of a single colony was transferred onto the MALDI target plate, overlaid with 1 µL of 70% formic acid (Sigma‐Aldrich), and allowed to air dry before the matrix solution was added. Results were analyzed using Bruker Daltonics flexControl 3.4 and MBT Compass Version 4.1 (library version 100). Each measurement was performed in duplicate for each culture and preparation. *Escherichia coli* DH5α was used as a quality control strain in each run. Identification as *S. mutans* was accepted when the MALDI‐TOF score was ≥ 2.0, indicating a reliable species‐level match according to the manufacturer's criteria.

### 2.2 Genome Sequencing

DNA from strains identified as *S. mutans* was extracted using the GenElute Bacterial Genomic DNA Kit. Complete genomic sequencing of the strains was performed on the Genome‐Transcriptome platform in Bordeaux using Illumina MiSeq technology with paired reads of 2 × 300 bp. Sequences were trimmed using Trimmomatic, assembled using Skesa, and annotated through the NCBI Prokaryotic Genome Annotation Pipeline (PGAP). The complete genomes of the 44 strains used in this study are available under the Bioproject accession number: PRJNA853131. Genome completeness, contamination levels, and number of contigs per assembly were assessed using CheckM version 1.2.3 (Parks et al. 2015).

### 2.3 In Silico Analyses

#### 2.3.1 Phylogenetic Trees

Two separate phylogenetic trees were generated using distinct approaches. The first was based on whole‐genome comparisons calculated through the Average Nucleotide Identity method (ANIm), which measures nucleotide‐level similarity by aligning genomes with MUMmer via the pyani tool v0.2.12 (https://github.com/widdowquinn/pyani) (Marcais et al. 2018; Pritchard et al. 2016). A custom Python script was used to convert the resulting similarity matrix into a distance matrix compatible with MEGA software (Kumar et al. 2018). The tree was reconstructed and visualized in iTOL (Letunic and Bork 2024), with CRISPR‐Cas system presence/absence data mapped alongside each branch.

The second tree was constructed from full‐length Cas9 protein sequences and their annotated domains, as identified by InterPro (Blum et al. 2025). Multiple sequence alignment was performed using Clustal Omega (Sievers and Higgins 2021) without trimming, and no specific gap‐handling was required due to the uniform size of the proteins and domains. Phylogenetic inference was conducted using the maximum likelihood method implemented in PhyML (Gabler et al. 2020; Guindon et al. 2009), and the final tree was visualized and annotated using iTOL.

### 2.4 Defense Systems Identification

The DefenseFinder tool version 1.2.2 (Tesson et al. 2022) and PADLOC version 2.0.0 (Payne et al. 2022) were used to identify known antiviral defense systems among the 44 sequenced genomes and in all publicly available *S. mutans* genomes retrieved from the NCBI GenBank Assembly database on May 17, 2024. Genomes were downloaded in FASTA format and the same detection pipeline was applied uniformly to all genomes.

### 2.5 CRISPR‐Cas Systems Analysis

To identify and characterize CRISPR‐Cas systems, genome sequences were analyzed using CRISPRCasFinder (Couvin et al. 2018). This tool was used to detect CRISPR arrays and classify the associated Cas genes into subtypes. Domains of Cas9 were predicted using InterPro (Blum et al. 2025) and multiple sequence alignment was performed using Clustal Omega (Gabler et al. 2020; Sievers and Higgins 2021).

### 2.6 Prophage Identification

Putative prophage‐like elements were identified using the PhiSpy pipeline (Akhter et al. 2012) integrated into the RAST server (Aziz et al. 2008; Brettin et al. 2015; Overbeek et al. 2014), which offers an automated platform for phage genome detection and annotation. Manual curation was subsequently performed to confirm the presence of hallmark sequences, including integrase genes, terminases, and genes encoding structural viral proteins. Prophage completeness and contamination were further evaluated using CheckV version 1.0.3 (Nayfach et al. 2021).

### 2.7 Direct Repeats (DRs) and Spacers in CRISPR‐Cas Systems

DRs and spacers within the CRISPR arrays were detected and characterized using CRISPRStudio (Dion et al. 2018) and CRISPRDetect (Biswas et al. 2016). DRs were analyzed to evaluate sequence conservation within the CRISPR‐Cas system. Spacers were aligned against the Core nucleotide database using Basic Local Alignment Search Tool (BLAST) to identify potential homologs and infer their origins. Shared spacers between strains were mapped to provide an overview of the most frequently observed sequences.

### 2.8 Anti‐CRISPR Detection

Anti‐CRISPR (Acr) elements were identified and analyzed using AcrFinder (Yi et al. 2020) and AcrHub (Wang et al. 2021). Identified Acr sequences were compared with reference Acr sequences by aligning them on the MultAlin platform (Corpet 1988).

## 3 Results

### 3.1 Defense Systems in *S. mutans*

CheckM evaluation of the genome quality of the 44 clinical *S. mutans* isolates analyzed in this study revealed completeness values never below 95% and a maximum contamination level of 2.34% (Table S1).

**FIGURE 1:**
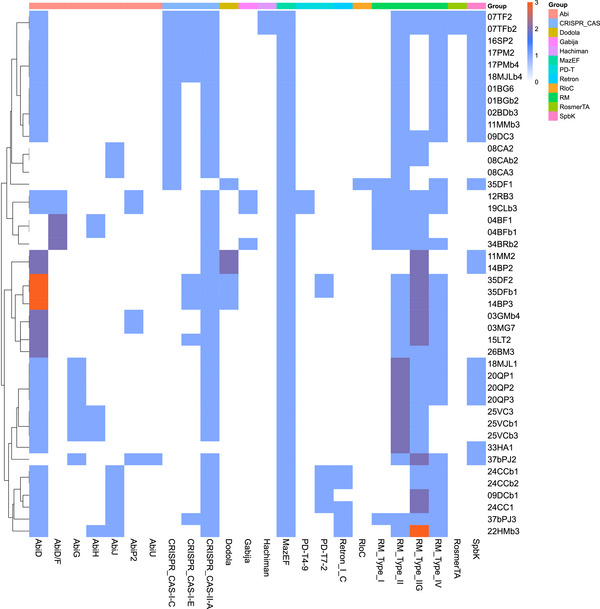
Antiphage Defense Systems identified in clinical isolates of *S. mutans*. The heatmap shows the presence and frequency of antiphage defense systems in *S. mutans* strains. Each row corresponds to a bacterial strain and each column represents a specific defense system. The relative frequency of each system within a given strain is color‐coded in proportion to its prevalence.

Each strain harbored between six and twelve APDSs (Figure 1). PADLOC and DefenseFinder identified twelve different APDS families across the 44 clinical *S. mutans* isolates sequenced in this study: “Abi”, CRISPR‐Cas, Dodola, Gabija, Hachiman, MazEF, a recently described family called PD (phage defense) (Vassallo et al. 2022), Retron, RloC, Restriction Modification (RM), RosmerTA, and Spbk. RM systems are the most prevalent, with Types I, II, IIG, and IV detected in our genomes, sometimes all coexisting within a single strain (e.g., 12RB3, 19CLb3, and 34BRb2). All strains have at least one RM system. The MazEF toxin–antitoxin system is also present in all strains. The CRISPR‐Cas and “Abi systems” follow in abundance. Three CRISPR‐Cas subtypes are observed: Subtypes I‐C and I‐E for class 1, and subtype II‐A for class 2. The “Abi systems” include members of the AbiD/F group (detected in DefenseFinder as Abi2), AbiG, AbiH, AbiJ, AbiP2, and AbiU. Other systems show variable distribution among the strains, with RloC (restriction‐linked ORF), Hachiman and RosmerTA being the least represented. The latter two are found in only two strains (07TF2 and 07TFb2), while RloC is exclusively present in strain 35DF1. Interestingly, 35DF1 is the only strain lacking an “Abi system”. The PD, Retron I‐C, Dodola and Gabija systems are present in 3 to 8 strains, while the Spbk system is found in twenty strains, including the Abi‐deficient strain 35DF1. This distribution pattern, observed in the 44 clinical isolates, reflects a broader diversity also found among other *S. mutans* genomes. These isolates are evenly distributed across the phylogeny of all publicly available *S. mutans* genomes (*n* = 478), of which they are a subset, indicating that the diversity in APDS content is not confined to a particular lineage. Consistent with this, no single APDS was found to be universally conserved across the species (Figure S2). Among the 39 distinct APDSs identified across the 478 publicly available *S. mutans* genomes including the 44 clinical isolates analyzed in this study, seven were overrepresented, together accounting for more than two‐thirds of all detected systems. Notably, four of them (AbiD, RM IIG, RM II, and MazEF) alone represented 40% of the total.

### 3.2 CRISPR Systems and Cas9 in *S. mutans*

**FIGURE 2:**
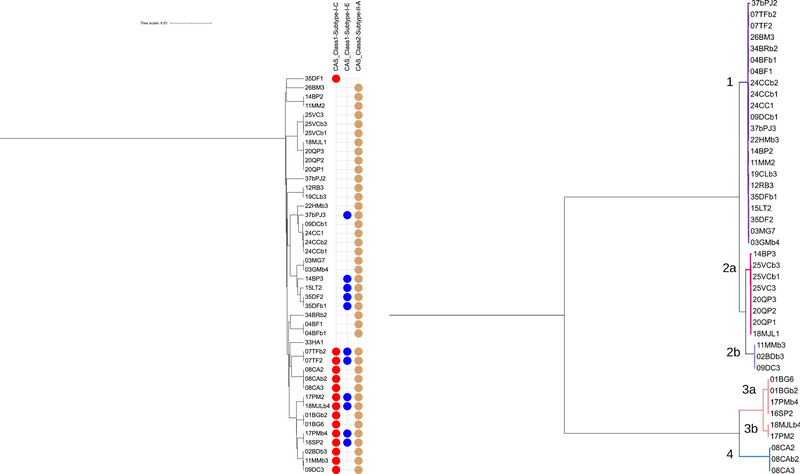
Phylogenetic Trees of CRISPR‐Cas Systems and Cas9 Proteins in *S. mutans*. **(A)** Phylogenetic tree of *S. mutans* strains. Subtypes of the CRISPR‐Cas systems retrieved (II‐A, I‐E, and I‐C) are indicated at the top of the figure. The presence of one or more subtypes in a strain is represented by colored circles adjacent to the strain's name: Beige for subtype II‐A, blue for subtype I‐E, and red for subtype I‐C. **(B)** Phylogenetic tree of Cas9 proteins from strains with type II‐A CRISPR‐Cas systems. Clustering highlights the sequence variations and classification of Cas9 proteins into distinct groups.

Genomic analysis of the 44 *S. mutans* strains revealed 68 CRISPR systems (Figure 2A). Almost all of these strains (95%) possess at least the CRISPR type II‐A system, with 52% possessing only this system. The type I‐C system is most commonly found in combination with type II‐A, either alone or together with type I‐E. In our study, type I‐C is never found with type I‐E alone, and it is the only CRISPR system present in strain 35DF1. However, type I‐E is always associated with either type II‐A alone or both types II‐A and I‐C. Notably, strain 33HA1 is unique in that it entirely lacks a CRISPR system. The presence of sequences that share high identity with cas genes in strain 37bPJ3 suggests the possible existence of a type I‐A system remnant in this strain.

The *cas9* genes of the 42 type II‐A systems were translated in silico and compared using Clustal Omega. This allowed for the classification of the Cas9 proteins into four distinct groups (Figure 2B). Group 1 and Group 2 share a similar domain structure, consisting of a REC lobe, HNH endonuclease, RuvC nuclease and PAM (protospacer adjacent motif) interacting (PI) domain. As previously demonstrated, whereas the PI domains of the two groups are very different, the other domains are very similar (Mosterd and Moineau 2020).

Group 1 is the largest group (22 members, 52 % of all found Cas9 proteins) and consists of members that are all 1345 aa in size and share near identity (>98 %) over the entire protein. They are very close (>98 %) to Cas9 of the well characterized system of *S. mutans* UA159 (van der Ploeg 2007, 2009). Group 2 members (11, 26 % of all Cas9 proteins) can be further subdivided into two subgroups: 2a (7/11, 1350 aa each) and 2b (4/11, 1339 aa). The Group 2a Cas9 proteins are identical to that of the well characterized system of *S. mutans* P42S (Mosterd and Moineau 2020, 2021). Group 2a and 2b have identical PI domains. Group 2a and 2b differentiate from each other mainly by their RuvC domain (75.32 % identity when comparing 18MJL1 from 2a and 02BDb3 from 2b). The RuvC domain of Group 2b is in fact nearly identical (>98 %) to that of Group 1. The REC lobe and HNH domain are more conserved between the two subgroups (88.18 % and 90.54 % identity, respectively).

Members of Group 3 and 4 are smaller and in addition to an alpha‐helical lobe, HNH and RuvC nucleases and PI domain, they possess a WED domain. Their hypothesized PI domain is smaller than that of Group 1 and 2 members. Group 3 (6 members, 14 % of all Cas9 proteins) can be further divided into two subgroups, 3a (4/6, 1125 aa) and 3b (2/6, 1126 aa), each with identical members. Group 3a and 3b share 92.25 % identity over the entire protein. When comparing only the PI domain, identity between Group 3a and 3b drops to 67.21 %. Group 4 consists of three members (7 % of all Cas9 proteins) that are identical in sequence and 1134 aa in size. Although Group 3 and 4 members share the same domain structure, they are different from each other on sequence level (67.14 % identity between Group 3a and 4, 67.03 % between 3b and 4). When comparing to Group 1 and 2, sequence identity does not exceed 21 % (Group 3) and 23 % (Group 4), respectively.

The different Cas9 proteins of *S. mutans* share similarity to Cas9 proteins from *S. thermophilus*. Group 1 and 2 members are similar to Cas9 CR3 and Group 3 and 4 members are similar to Cas9 CR1 in size, domain structure and sequence (sequence identity between CR3 and Group 1 and 2 members ranges from 61% to 63 % and between CR1 and Group 3 and 4 from 64% to 71 %).

### 3.3 Prophage Presence in *S. mutans*

**FIGURE 3:**
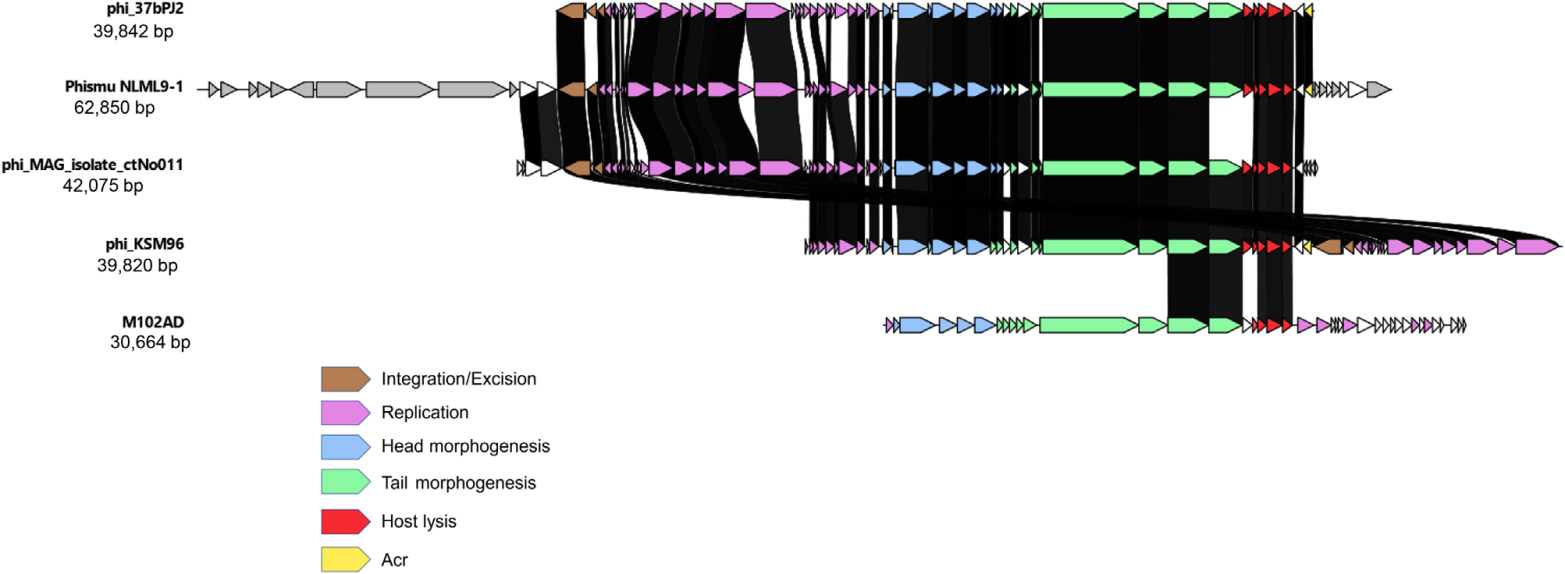
Comparison of Φ37bJ2 with other bacteriophages infecting *S. mutans*. The Φ37bJ2 sequence (JANDZM010000008.1) was compared with the sequence of the *Streptococcus mutans* temperate bacteriophage ΦKSM96 (OQ627164) isolated by Sugai et al., two previously sequenced prophages phi_MAG_isolate_ctNo011 (BK034220.1) and Phismu NLML9‐1 (AHSJ01000002.1) and the virulent bacteriophage M102AD (DQ386162). Homologous genes are indicated based on nucleotide sequence similarity, with a high consensus threshold set at 90% identity and a low consensus threshold at 50% identity.

Using the PhiSpy pipeline of RAST, we identified an intact prophage in strain 37bPJ2, which from hereon is called phi_37bPJ2 (Φ37bPJ2). The complete annotated genomic sequence of this phage is available in GenBank under accession no. JANDZM010000008.1. CheckV analysis confirmed the high quality of this prophage genome, with 100% completeness and no contamination. The sequence of phi_37bPJ2 was compared to other *S. mutans* phages, which revealed a strong resemblance (93.21 %) to prophage ctNo011 (Figure 3). Genomic regions encoding essential genes were highly conserved between the analyzed phages.

Conserved genes shared by both lytic phages and prophages exhibited sequence identities above 90%, including the tail tape measure protein (TMP) at 96%–98%, the small terminase at 99%, the large terminase at 92%–98%, the two endolysins at 92%, and the two holins at 90%–92%. Notably, the integrase, a gene specific to temperate phages, showed a high level of conservation with 97% sequence identity.

### 3.4 Spacers and DRs in CRISPR Systems of *S. mutans*

**FIGURE 4:**
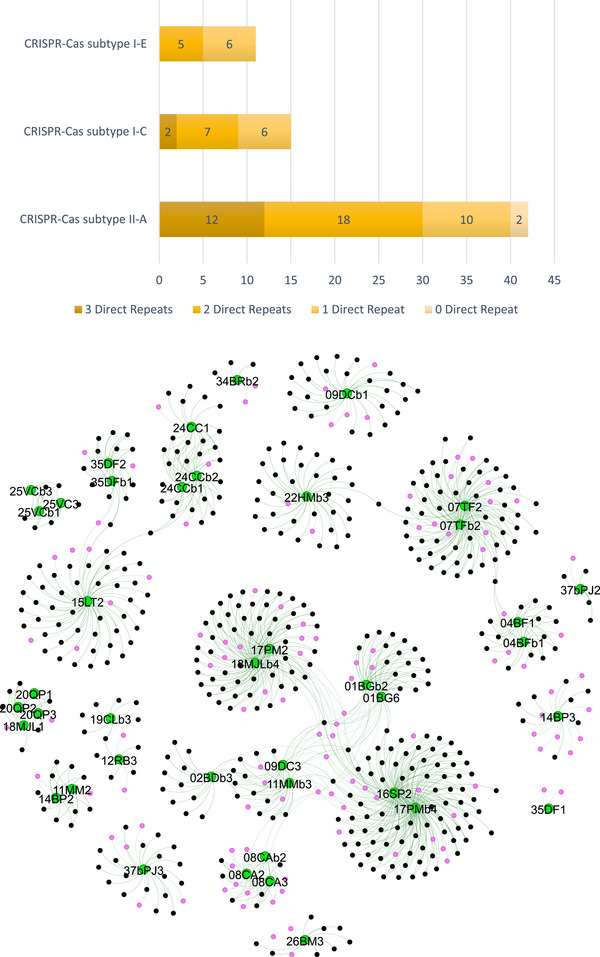
Analysis of Direct Repeat and Spacers in *S. mutans* CRISPR‐Cas systems. **(A)** Stacked bar chart illustrating the distribution of direct repeat (DR) configurations within CRISPR arrays across the three described subtypes (I‐E, I‐C, and II‐A). The bars represent the number of CRISPR arrays with 3, 2, 1, or 0 direct repeats, providing a comparative overview of DR configurations by subtype. **(B)** Network diagram showing the number of spacers identified per strain and between which strains they are shared. Shared spacers are connected by edges, while spacers with BLAST homology are highlighted in pink.

Of all the DRs identified in this study, 62% are associated with CRISPR‐Cas type II‐A (Figure 4A). The number of different DR sequences per CRISPR system varies, with a maximum of three observed. In seven strains, variations in DRs for a given CRISPR‐Cas system include differences in the first four nucleotides (see Table S3 for detailed sequences). The most common configuration was two different DRs per system, representing 44% of the total. Two strains, 03MG7 and 03GMb4, possess a type II‐A CRISPR system without an associated CRISPR locus. Strain 37bPJ3 appears to carry remnants of a type I‐A system, with no associated CRISPR locus retrieved.

In contrast, the remaining 41 strains with an apparently complete CRISPR system contain a total of 1059 spacers, of which 579 are unique (Figure 4B). BLAST analysis revealed homologous sequences for 117 spacers targeting different nucleotide sequences encoding terminases, TMPs, or endolysins, among others (see Table S4, for details). Notably, approximately 80% of the spacers corresponded to “dark matter” sequences, for which no identifiable matches were found.

### 3.5 Anti‐CRISPR Protein of the Phage phi_37bPJ2

**FIGURE 5:**
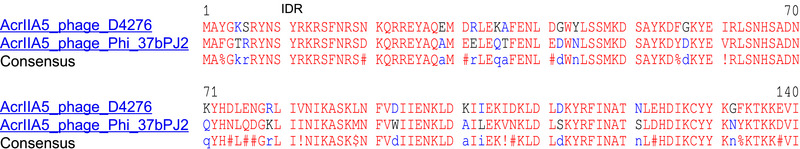
Anti‐CRISPR AcrIIA5 protein identified in the phi_37bPJ2 phage. Alignment comparing the AcrIIA5 protein from prophage phi_37bPJ2 with the AcrIIA5 protein encoded by the *S. thermophilus* prophage D4276. The intrinsically disordered region (IDR) at the N‐terminus, is highlighted. The alignment was performed using the Multalin tool (http://multalin.toulouse.inra.fr/multalin).

Through sequence analysis via AcrFinder and AcrHub, we identified an anti‐CRISPR (Acr) sequence in phi_37bPJ2. The identified sequence encodes an AcrIIA5‐type Acr (GenBank accession number MDT9502488.1). Sequence identity with AcrIIA5 of lytic phage D4276 targeting *S. thermophilus* is 76% (Figure 5). At the N‐terminus of the AcrIIA5 of phi_37bPJ2, there is a 22 amino acid segment known as the Intrinsically Disordered Region (IDR), which tends to be positively charged containing four arginine and two lysine residues. Sequence identity of this Acr with Acr from other prophages infecting *S. mutans* is over 90% (Figure S5).

## 4 Discussion

In this study, we identified multiple APDSs in clinical isolates of *S. mutans*, with each strain carrying between six and twelve systems. In addition, we identified a prophage in one of the strains, named phi_37bPJ2, which encodes an anti‐CRISPR protein (AcrIIA5).

### 4.1 Defense Systems in *S. mutans*

Our study systematically mapped previously described APDSs in 44 newly sequenced clinical isolates of *S. mutans*, which were analyzed alongside 434 other publicly available genomes (total *n* = 478). Many of these systems have only recently been characterized in other bacterial species and had not yet been documented in *S. mutans*. As a result, the diversity and genomic organization of APDSs in this species remain poorly understood.

Restriction‐modification (RM) systems were the most prevalent across the clinical isolates, followed by the MazEF toxin–antitoxin and CRISPR‐Cas systems. CRISPR‐Cas systems are discussed in the following paragraph. The main studies mentioning RM systems refer only to the type II RM system (Banas et al. 2011; Zhao et al. 2024). The study by Argimon et al., which also mentions RM systems, does not specify their type (Argimon et al. 2014). The RloC system has already been reported in *S. mutans* and is considered a distant homolog of the PrrC anticodon nuclease (ACNase), with putative antiphage activity. It carries a conserved ACNase domain and a zinc‐hook motif reminiscent of DNA repair proteins. According to Davidov et al., this system is systematically associated with the type I‐C RM system (Davidov and Kaufmann 2008). In our study, the only strain with RloC was found to have a type I RM system. In this strain, we also found the Spbk system, which according to the work of Johnson et al., acts as an abortive infection system (Johnson et al. 2022). The MazEF type II toxin–antitoxin system has been previously characterized and shown to function as a growth modulator under adverse environmental conditions (Lemos et al. 2005; Syed et al. 2011). As a bacterial defense mechanism, it promotes cell death in response to stress or phage infection, thereby limiting phage propagation and enhancing population‐level survival (Ramisetty et al. 2015).

Comparative data on other defense systems in *S. mutans* remain limited, both because of their recent discovery and the lack of comprehensive analyses across multiple strains.

However, Kelleher and colleagues recently demonstrated the presence of the Gabija, Dodola, and “Abi systems” in *Streptococcus thermophilus*, in addition to the CRISPR‐Cas and RM systems, which appear to be the most important (Kelleher et al. 2024). In their study, and in contrast to our findings, the defense systems are essentially clustered in “phage defense islands”. Although defense systems are commonly associated with clustering, the presence of defense islands also appeared to be absent in a study performed using *Lactococcus* species (Grafakou et al. 2024). More recently, a large‐scale genomic analysis of 263 *S. thermophilus* strains identified 21 distinct non‐CRISPR, non‐RM defense systems, with AbiD being the most prevalent and most others detected in less than 15% of strains (Leprince et al. 2025). These findings further emphasize the possible variability of defense repertoires across streptococci.

To ensure the robustness of our analysis, it is worth noting that the genomes sequenced in this study were of high quality, as confirmed by CheckM: All genomes showed completeness values above 95% and contamination levels below 5%, thus qualifying as high‐quality draft genomes (Bowers et al. 2017). This level of quality supports the reliability of the observed distribution and organization of APDSs across *S. mutans* strains.

Nevertheless, the detection of certain systems, particularly those involving repetitive elements such as CRISPR arrays or prophages, can still be hindered by the use of short‐read sequencing technologies like Illumina. These methods often struggle to resolve long or repetitive genomic regions, which may lead to incomplete assemblies and an underestimation of the true diversity and architecture of defense elements (Heydari et al. 2019; Stoler and Nekrutenko 2021). Although all 44 clinical isolates were obtained from people with Parkinson's disease, they are phylogenetically well distributed within the 478‐genome dataset to which they belong, and were collected from both caries‐active and caries‐free participants (Donnet et al. 2025). In light of this, no conclusions were drawn regarding the clinical background of the donors, as additional contextual factors, such as comorbidities, smoking status, or socioeconomic background, would be required to support such an analysis. Furthermore, such metadata are not available for the publicly available genomes.

### 4.2 CRISPR Systems and Cas9 in *S. mutans*

As observed in other *Streptococcus* species relevant to human and animal health, the type II‐A CRISPR‐Cas system is the most prevalent in *S. mutans* (Lemaire et al. 2022). While type I‐C and I‐E systems are also present, as previously described for *S. mutans* (Lemaire et al. 2022; Serbanescu et al. 2015; van der Ploeg 2009), no type II‐C was found. The frequency of this subtype had already been documented as lower than the other three mentioned above (Lemaire et al. 2022). We also identified one *S. mutans* strain that completely lacks a CRISPR‐Cas system. A similar observation has been made in *Streptococcus pyogenes*, where strains without CRISPR‐Cas carried significantly more prophages than those with it (Nozawa et al. 2011). Here, the only strain lacking CRISPR‐Cas is also not lysogenic. This observation raises questions about the role of other defense systems in combating prophage integration.

In a previous study of Cas9 diversity in *S. mutans* type II‐A systems, two main groups were described with conserved catalytic domains but highly variable PI domains (Mosterd and Moineau 2020). These same groups are present in our 44 strains (here named Group 1 and 2a), but several additional groups and subgroups have now been found. Group 3 and 4 consist of members that are smaller in size and with a domain structure different from what has been described in *S. mutans*. Their PI domains are different from those of characterized Cas9 proteins. Where Group 1 members recognize NGG as PAM (van der Ploeg 2009) and Group 2 members recognize NAA or NGAA (Mosterd and Moineau 2020, 2021), the PAMs targeted by Group 3 and 4 Cas9 proteins are unknown. This invites experimental validation to obtain the full picture of the PAMs recognized by *S. mutans* type II‐A systems.

Most streptococcal type II‐A CRISPR‐Cas systems were found to be homologous to CR3 from *S. thermophilus* and CR1 homologues are rare (Lemaire et al. 2022). Previously, only CR3 homologues were described for *S. mutans* (Group 1 and 2), but with the discovery of Group 3 and 4, we demonstrate that 9 out of the 42 (21 %) type II‐A systems among our *S. mutans* strains are in fact CR1 homologues. Under laboratory conditions, *S. thermophilus* CR1 is almost one tenfold more active than CR3 (Magadan et al. 2012).

### 4.3 Prophage Presence

So far, only five phages infecting *S. mutans* have been isolated and fully sequenced to allow complete characterization: Four lytic phages (M102, M102AD, ΦAPCM01, and SMHBZ8) and one temperate phage (phi_KSM96) recently described by Sugai et al. In contrast, two additional lytic phages (e10 and f1) were reported by Delisle in 1993, but they remain unsequenced (Delisle and Rostkowski 1993). Data on prophages in *S. mutans* are similarly sparse. Fu et al. identified 35 prophages by in silico analysis of 171 *S. mutans* genomes, but most were incomplete, with only three having complete sequences (phismuNLML9‐1, phismuN66‐1, and phismu24‐1) (Fu et al. 2017). Earlier, in 1977, Higuchi and colleagues reported the presence of a phage in the lysogenic *S. mutans* PK1 strain, but no genomic characterization was performed (Higuchi et al. 1977).

Sequence analysis of the strains in this study identified a unique prophage, phi_37bPJ2, which shares high sequence identity with two previously described phages: The ctNo011 prophage recovered from a human metagenome (Tisza and Buck 2021) and the phi_KSM96 phage isolated from *S. mutans* (Sugai et al. 2023). Notably, ctNo011 was initially assigned to the genus *Streptococcus* in general rather than specifically to *S. mutans*. Among the three phages, the sequence similarity is particularly pronounced in their integrase genes, suggesting that they all share the same integration site within the *S. mutans* genome. According to Sugai et al., this integration site is located between the comGB and comGC genes (or comYB and comYC, respectively, in the *S. mutans* UA159 genome) (Sugai et al. 2023). It should be noted that the contig carrying the phismuNLML9‐1 phage also contains the comGC gene (data not shown). The contiguity of the two holins and two endolysins in the phi_37bPJ2 genome is consistent with the typical organization of a lysis module. This arrangement aligns with previous findings on the lysis module structure of phages M102, M102AD, and ΦAPCM01 (Saint‐Jean et al. 2025).

### 4.4 Spacers and DR in CRISPR Systems of *S. mutans*

DRs within a single CRISPR array are typically highly conserved (Jansen et al. 2002). However, variations have been observed in certain bacterial species, often limited to the final DR of the array, which is commonly described as degenerate (Biswas et al. 2016). In this study, some strains exhibited not only variations among DRs within the same CRISPR array but also differences in the first four nucleotides of the DR sequence. Such mutations in the initial nucleotides have previously been shown to affect spacer integration efficiency, potentially reducing its effectiveness (Grainy et al. 2019).

Among the spacer targets identified in this study, the majority fall into the category of “dark matter,” which refers to sequences whose function or origin remains unknown because they do not match any currently known genomes or mobile genetic elements in existing databases. The proportion of “dark matter” spacers observed in our study is consistent with the results reported by Rubio et al. (2023). They introduced an innovative strategy to reduce dark matter by constructing a pangenome (a comprehensive set of genes present in all genomes of a species), which significantly reduced the proportion of unidentified spacers. Notably, most of the spacers in their analysis targeted species‐specific membrane proteins. In contrast, Shmakov and colleagues primarily identified spacers targeting species‐specific mobile genetic elements, including the virome (Shmakov et al. 2017, 2020). These two perspectives reflect the current main hypotheses regarding the origin of spacers: the mobilome and the host genome itself (autoimmunity), aligning with the conclusions of Walker and Shields (2022).

### 4.5 Anti‐CRISPR (Acr) Protein of the Phage phi_37bPJ2

In their ongoing arms race with bacteria, phages have evolved multiple strategies to inactivate CRISPR‐Cas systems, including the development of over 300 experimentally confirmed anti‐CRISPR proteins (Acrs) (Wang et al. 2021). However, the efficacy of these Acrs can vary widely. For example, Garcia et al. showed that out of ten anti‐CRISPR proteins tested, only AcrIIA5 effectively inhibited all type II‐A and II‐C Cas9 proteins tested (Garcia et al. 2019). Further research by An and colleagues suggested that AcrIIA5‐mediated inhibition relies on a critical 22‐amino acid segment at the N‐terminus known as the IDR. Maintaining the full length of this IDR is critical for its activity, as any reduction in size significantly impairs or eliminates its ability to inhibit Cas9 (An et al. 2020). Unlike the AcrIIA5 found in the virulent phage D4276 that infects *S. thermophilus*, the IDR of the one of phi_37bPJ2 contains only six arginine or lysine residues instead of seven. These residues are critical for Acr function, and in particular the positively charged cluster of Arg12, Lys13, and Arg14 has been shown to play a key role in Cas9 inhibition (An et al. 2020). In our study, this positively charged region is well conserved in the AcrIIA5 of phi_37bPJ2. Combined with its integration into strain 37bPJ2, these findings strongly support the functional activity of AcrIIA5 in phi_37bPJ2.

The results of this study provide new insights into the diversity of APDSs in *S. mutans* and their potential role in bacterial adaptation to phage pressure. Future research should explore the functional significance of these systems, particularly CRISPR‐Cas, in light of the recently identified diversity of Cas9 proteins in *S. mutans* and the frequent occurrence of AcrIIA5 in its infecting prophages. Extending genomic and functional analyses to additional clinical isolates may further elucidate the evolutionary dynamics shaping these defense systems.

## Author Contributions

**Olivier Claisse**: data curation, formal analysis, conceptualization, methodology, writing – original draft, writing – review and editing. **Cas Mosterd**: formal analysis, conceptualization, methodology, writing – original draft, writing – review and editing. **Claire Le Marrec**: conceptualization, formal analysis, writing – review and editing. **Johan Samot**: project administration, conceptualization, methodology, funding acquisition, investigation, formal analysis, validation, writing – original draft, writing – review and editing.

## Funding

This study was supported in part by France Parkinson.

## Conflicts of Interest

The authors declare no conflicts of interest.

## Supporting information




**Table S1**: CheckM genome quality report for the 44 sequenced *Streptococcus mutans* strains.

**Figure S2(a)**: Overview of antiphage defense systems (APDSs) identified in the genomes of 478 publicly available *S. mutans* strains (data retrieved in May 2024).

**Figure S2(b)**: APDSs identified in the genomes of 44 newly sequenced *S. mutans* clinical isolates.

**Figure S2(c)**: Phylogenetic placement of the 44 clinical isolates within the full set of 478 *S. mutans* genomes. Clinical isolates are marked by black circles and labeled with their strain names. The phylogenetic tree was rooted using *Streptococcus anginosus* J4211 as an outgroup, given its close yet distinct taxonomic relationship to *S. mutans*.

**Table S3**: CRISPR‐Cas systems and their associated direct repeats in the 44 clinical isolates of *S. mutans* from this study.

**Table S4**: Overview of BLAST matches for 117 spacers out of 579 unique sequences: associated bacterial strains and phages.

**Figure S5**: Alignment of the AcrIIA5 protein from prophage phi_37bPJ2 with AcrIIA5 proteins from other phages infecting *Streptococcus mutans*.

## Data Availability

All data generated or analyzed during this study are included in this article and its supplementary material files. Further enquiries can be directed to the corresponding author.

## References

[omi70014-bib-0001] Akhter, S. , R. K. Aziz , and R. A. Edwards . 2012. “PhiSpy: A Novel Algorithm for Finding Prophages in Bacterial Genomes That Combines Similarity‐ and Composition‐Based Strategies.” Nucleic Acids Research 40, no. 16: e126. 10.1093/nar/gks406. 22584627 PMC3439882

[omi70014-bib-0002] Al‐Jarbou, A. N. 2012. “Genomic Library Screening for Viruses From the Human Dental Plaque Revealed Pathogen‐Specific Lytic Phage Sequences.” Current Microbiology 64, no. 1: 1–6. 10.1007/s00284-011-0025-z. 21969025

[omi70014-bib-0003] An, S. Y. , D. Ka , I. Kim , et al. 2020. “Intrinsic Disorder Is Essential for Cas9 Inhibition of Anti‐CRISPR AcrIIA5.” Nucleic Acids Research 48, no. 13: 7584–7594. 10.1093/nar/gkaa512. 32544231 PMC7367191

[omi70014-bib-0004] Argimon, S. , K. Konganti , H. Chen , A. V. Alekseyenko , S. Brown , and P. W. Caufield . 2014. “Comparative Genomics of Oral Isolates of *Streptococcus mutans* by in Silico Genome Subtraction Does Not Reveal Accessory DNA Associated With Severe Early Childhood Caries.” Infection, Genetics and Evolution 21: 269–278. 10.1016/j.meegid.2013.11.003. PMC3940162 24291226

[omi70014-bib-0005] Aziz, R. K. , D. Bartels , A. A. Best , et al. 2008. “The RAST Server: Rapid Annotations Using Subsystems Technology.” BMC Genomics 9: 75. 10.1186/1471-2164-9-75. 18261238 PMC2265698

[omi70014-bib-0006] Banas, J. A. , S. Biswas , and M. Zhu . 2011. “Effects of DNA Methylation on Expression of Virulence Genes in *Streptococcus mutans* .” Applied and Environmental Microbiology 77, no. 20: 7236–7242. 10.1128/AEM.00543-11. 21841035 PMC3194855

[omi70014-bib-0007] Ben‐Zaken, H. , R. Kraitman , S. Coppenhagen‐Glazer , et al. 2021. “Isolation and Characterization of *Streptococcus mutans* Phage as a Possible Treatment Agent for Caries.” Viruses 13, no. 5: 825. 10.3390/v13050825. 34063251 PMC8147482

[omi70014-bib-0008] Biswas, A. , R. H. Staals , S. E. Morales , P. C. Fineran , and C. M. Brown . 2016. “CRISPRDetect: A Flexible Algorithm to Define CRISPR Arrays.” BMC Genomics 17: 356. 10.1186/s12864-016-2627-0. 27184979 PMC4869251

[omi70014-bib-0009] Blum, M. , A. Andreeva , L. C. Florentino , et al. 2025. “InterPro: The Protein Sequence Classification Resource in 2025.” Nucleic Acids Research 53, no. D1: D444–D456. 10.1093/nar/gkae1082. 39565202 PMC11701551

[omi70014-bib-0010] Bowers, R. M. , N. C. Kyrpides , R. Stepanauskas , et al. 2017. “Minimum Information About a Single Amplified Genome (MISAG) and a Metagenome‐Assembled Genome (MIMAG) of Bacteria and Archaea.” Nature Biotechnology 35, no. 8: 725–731. 10.1038/nbt.3893. PMC6436528 28787424

[omi70014-bib-0011] Brettin, T. , J. J. Davis , T. Disz , et al. 2015. “RASTtk: A Modular and Extensible Implementation of the RAST Algorithm for Building Custom Annotation Pipelines and Annotating Batches of Genomes.” Scientific Reports 5: 8365. 10.1038/srep08365. 25666585 PMC4322359

[omi70014-bib-0012] Corpet, F. 1988. “Multiple Sequence Alignment With Hierarchical Clustering.” Nucleic Acids Research 16, no. 22: 10881–10890. 10.1093/nar/16.22.10881. 2849754 PMC338945

[omi70014-bib-0013] Couvin, D. , A. Bernheim , C. Toffano‐Nioche , et al. 2018. “CRISPRCasFinder, an Update of CRISRFinder, Includes a Portable Version, Enhanced Performance and Integrates Search for Cas Proteins.” Nucleic Acids Research 46, no. W1: W246–W251. 10.1093/nar/gky425. 29790974 PMC6030898

[omi70014-bib-0014] Dalmasso, M. , E. de Haas , H. Neve , et al. 2015. “Isolation of a Novel Phage With Activity Against *Streptococcus mutans* Biofilms.” PLoS ONE 10, no. 9: e0138651. 10.1371/journal.pone.0138651. 26398909 PMC4580409

[omi70014-bib-0015] Davidov, E. , and G. Kaufmann . 2008. “RloC: A Wobble Nucleotide‐Excising and Zinc‐Responsive Bacterial tRNase.” Molecular Microbiology 69, no. 6: 1560–1574. 10.1111/j.1365-2958.2008.06387.x. 18681940 PMC2610378

[omi70014-bib-0016] Delisle, A. L. , M. Guo , N. I. Chalmers , G. J. Barcak , G. M. Rousseau , and S. Moineau . 2012. “Biology and Genome Sequence of *Streptococcus mutans* Phage M102AD.” Applied and Environmental Microbiology 78, no. 7: 2264–2271. 10.1128/AEM.07726-11. 22287009 PMC3302630

[omi70014-bib-0017] Delisle, A. L. , and C. A. Rostkowski . 1993. “Lytic Bacteriophages of *Streptococcus mutans* .” Current Microbiology 27, no. 3: 163–167. 10.1007/BF01576015. 23835749

[omi70014-bib-0018] Dion, M. B. , S. J. Labrie , S. A. Shah , and S. Moineau . 2018. “CRISPRStudio: A User‐Friendly Software for Rapid CRISPR Array Visualization.” Viruses 10, no. 11: 602. 10.3390/v10110602. 30388811 PMC6267562

[omi70014-bib-0019] Donnet, L. , O. Claisse , and J. Samot . 2025. “Serotype and Distribution of Adhesion Genes in *Streptococcus mutans* Isolated From People With Parkinson's Disease.” Odontology 113, no. 4: 1520–1532. 10.1007/s10266-025-01078-5. 40048130

[omi70014-bib-0020] Fu, T. , X. Fan , Q. Long , W. Deng , J. Song , and E. Huang . 2017. “Comparative Analysis of Prophages in *Streptococcus mutans* Genomes.” PeerJ 5: e4057. 10.7717/peerj.4057. 29158986 PMC5695247

[omi70014-bib-0021] Gabler, F. , S. Z. Nam , S. Till , et al. 2020. “Protein Sequence Analysis Using the MPI Bioinformatics Toolkit.” CP in Bioinformatics 72, no. 1: e108. 10.1002/cpbi.108. 33315308

[omi70014-bib-0022] Garcia, B. , J. Lee , A. Edraki , et al. 2019. “Anti‐CRISPR AcrIIA5 Potently Inhibits all Cas9 Homologs Used for Genome Editing.” Cell Reports 29, no. 7: 1739–1746.e5. 10.1016/j.celrep.2019.10.017. 31722192 PMC6910239

[omi70014-bib-0023] Grafakou, A. , C. Mosterd , M. H. Beck , et al. 2024. “Discovery of Antiphage Systems in the Lactococcal Plasmidome.” Nucleic Acids Research 52, no. 16: 9760–9776. 10.1093/nar/gkae671. 39119896 PMC11381338

[omi70014-bib-0024] Grainy, J. , S. Garrett , B. R. Graveley , and M. P. Terns . 2019. “CRISPR Repeat Sequences and Relative Spacing Specify DNA Integration by *Pyrococcus furiosus* Cas1 and Cas2.” Nucleic Acids Research 47, no. 14: 7518–7531. 10.1093/nar/gkz548. 31219587 PMC6698737

[omi70014-bib-0025] Guindon, S. , F. Delsuc , J. F. Dufayard , and O. Gascuel . 2009. “Estimating Maximum Likelihood Phylogenies With PhyML.” Methods in Molecular Biology 537: 113–137. 10.1007/978-1-59745-251-9_6. 19378142

[omi70014-bib-0026] Heydari, M. , G. Miclotte , Y. Van de Peer , and J. Fostier . 2019. “Illumina Error Correction near Highly Repetitive DNA Regions Improves De Novo Genome Assembly.” BMC Bioinformatics 20, no. 1: 298. 10.1186/s12859-019-2906-2. 31159722 PMC6545690

[omi70014-bib-0027] Higuchi, M. , M. Higuchi , and A. Katayose . 1982. “Identification of PK 1 Bacteriophage DNA in *Streptococcus mutans* .” Journal of Dental Research 61, no. 2: 439–441. 10.1177/00220345820610021501. 6948863

[omi70014-bib-0028] Higuchi, M. , G. H. Rhee , S. Araya , and M. Higuchi . 1977. “Bacteriophage Deoxyribonucleic Acid‐Induced Mutation of *Streptococcus mutans* .” Infection and Immunity 15, no. 3: 938–944. 10.1128/iai.15.3.938-944.1977. 870435 PMC421463

[omi70014-bib-0029] Jansen, R. , J. D. Embden , W. Gaastra , and L. M. Schouls . 2002. “Identification of Genes That Are Associated With DNA Repeats in Prokaryotes.” Molecular Microbiology 43, no. 6: 1565–1575. 10.1046/j.1365-2958.2002.02839.x. 11952905

[omi70014-bib-0030] Jie, Z. , H. Liang , Y. Meng , et al. 2025. “Integrating Metagenomics and Cultivation Unveils Oral Phage Diversity and Potential Impact on Hosts.” Npj Biofilms and Microbiomes 11, no. 1: 145. 10.1038/s41522-025-00773-z. 40715125 PMC12297358

[omi70014-bib-0031] Johnson, C. M. , M. M. Harden , and A. D. Grossman . 2022. “Interactions Between Mobile Genetic Elements: an Anti‐Phage Gene in an Integrative and Conjugative Element Protects Host Cells From Predation by a Temperate Bacteriophage.” Plos Genetics 18, no. 2: e1010065. 10.1371/journal.pgen.1010065. 35157704 PMC8880864

[omi70014-bib-0032] Kelleher, P. , G. Ortiz Charneco , Z. Kampff , et al. 2024. “Phage Defence Loci of *Streptococcus thermophilus*‐tip of the Anti‐Phage Iceberg?” Nucleic Acids Research 52, no. 19: 11853–11869. 10.1093/nar/gkae814. 39315705 PMC11514479

[omi70014-bib-0033] Kumar, S. , G. Stecher , M. Li , C. Knyaz , and K. Tamura . 2018. “MEGA X: Molecular Evolutionary Genetics Analysis Across Computing Platforms.” Molecular Biology and Evolution 35, no. 6: 1547–1549. 10.1093/molbev/msy096. 29722887 PMC5967553

[omi70014-bib-0034] Lemaire, C. , B. Le Gallou , P. Lanotte , L. Mereghetti , and A. Pastuszka . 2022. “Distribution, Diversity and Roles of CRISPR‐Cas Systems in Human and Animal Pathogenic Streptococci.” Frontiers in Microbiology 13: 828031. 10.3389/fmicb.2022.828031. 35173702 PMC8841824

[omi70014-bib-0035] Lemos, J. A. , T. A. Brown Jr. , J. Abranches , and R. A. Burne . 2005. “Characteristics of *Streptococcus mutans* Strains Lacking the MazEF and RelBE Toxin‐Antitoxin Modules.” Fems Microbiology Letters 253, no. 2: 251–257. 10.1016/j.femsle.2005.09.045. 16243456

[omi70014-bib-0036] Leprince, A. , J. Lefrancois , A. M. Millen , et al. 2025. “Strengthening Phage Resistance of *Streptococcus thermophilus* by Leveraging Complementary Defense Systems.” Nature Communications 16, no. 1: 7142. 10.1038/s41467-025-62408-3. PMC12322248 40759662

[omi70014-bib-0037] Letunic, I. , and P. Bork . 2024. “Interactive Tree of Life (iTOL) v6: Recent Updates to the Phylogenetic Tree Display and Annotation Tool.” Nucleic Acids Research 52, no. W1: W78–W82. 10.1093/nar/gkae268. 38613393 PMC11223838

[omi70014-bib-0038] Magadan, A. H. , M. E. Dupuis , M. Villion , and S. Moineau . 2012. “Cleavage of Phage DNA by the *Streptococcus thermophilus* CRISPR3‐Cas System.” PLoS ONE 7, no. 7: e40913. 10.1371/journal.pone.0040913. 22911717 PMC3401199

[omi70014-bib-0039] Marcais, G. , A. L. Delcher , A. M. Phillippy , R. Coston , S. L. Salzberg , and A. Zimin . 2018. “MUMmer4: A Fast and Versatile Genome Alignment System.” Plos Computational Biology 14, no. 1: e1005944. 10.1371/journal.pcbi.1005944. 29373581 PMC5802927

[omi70014-bib-0040] Millman, A. , S. Melamed , A. Leavitt , et al. 2022. “An Expanded Arsenal of Immune Systems That Protect Bacteria From Phages.” Cell Host & Microbe 30, no. 11: 1556–1569.e5. 10.1016/j.chom.2022.09.017. 36302390

[omi70014-bib-0041] Mosterd, C. , and S. Moineau . 2020. “Characterization of a Type II‐A CRISPR‐Cas System in *Streptococcus mutans* .” mSphere 5, no. 3: e00235–20. 10.1128/mSphere.00235-20. PMC7316486 32581075

[omi70014-bib-0042] Mosterd, C. , and S. Moineau . 2021. “Primed CRISPR‐Cas Adaptation and Impaired Phage Adsorption in *Streptococcus mutans* .” mSphere 6, no. 3: e00185–21. 10.1128/mSphere.00185-21. PMC8265633 34011685

[omi70014-bib-0043] Nayfach, S. , A. P. Camargo , F. Schulz , E. Eloe‐Fadrosh , S. Roux , and N. C. Kyrpides . 2021. “CheckV Assesses the Quality and Completeness of Metagenome‐assembled Viral Genomes.” Nature Biotechnology 39, no. 5: 578–585. 10.1038/s41587-020-00774-7. PMC8116208 33349699

[omi70014-bib-0044] Nozawa, T. , N. Furukawa , C. Aikawa , et al. 2011. “CRISPR Inhibition of Prophage Acquisition in *Streptococcus pyogenes* .” PLoS ONE 6, no. 5: e19543. 10.1371/journal.pone.0019543. 21573110 PMC3089615

[omi70014-bib-0045] Overbeek, R. , R. Olson , G. D. Pusch , et al. 2014. “The SEED and the Rapid Annotation of Microbial Genomes Using Subsystems Technology (RAST).” Nucleic Acids Research 42, no. D1: D206–D214. 10.1093/nar/gkt1226. 24293654 PMC3965101

[omi70014-bib-0046] Parks, D. H. , M. Imelfort , C. T. Skennerton , P. Hugenholtz , and G. W. Tyson . 2015. “CheckM: Assessing the Quality of Microbial Genomes Recovered From Isolates, Single Cells, and Metagenomes.” Genome Research 25, no. 7: 1043–1055. 10.1101/gr.186072.114. 25977477 PMC4484387

[omi70014-bib-0047] Payne, L. J. , S. Meaden , M. R. Mestre , et al. 2022. “PADLOC: A Web Server for the Identification of Antiviral Defence Systems in Microbial Genomes.” Nucleic Acids Research 50, no. W1: W541–W550. 10.1093/nar/gkac400. 35639517 PMC9252829

[omi70014-bib-0048] Pritchard, L. , R. H. Glover , S. Humphris , J. G. Elphinstone , and I. K. Toth . 2016. “Genomics and Taxonomy in Diagnostics for Food Security: Soft‐Rotting Enterobacterial Plant Pathogens.” Analytical Methods 8, no. 1: 12–24. 10.1039/c5ay02550h.

[omi70014-bib-0049] Ramisetty, B. C. , B. Natarajan , and R. S. Santhosh . 2015. “mazEF‐Mediated Programmed Cell Death in Bacteria: “What Is This?”.” Critical Reviews in Microbiology 41, no. 1: 89–100. 10.3109/1040841X.2013.804030. 23799870

[omi70014-bib-0050] Rubio, A. , M. Sprang , A. Garzon , et al. 2023. “Analysis of Bacterial Pangenomes Reduces CRISPR Dark Matter and Reveals Strong Association Between Membranome and CRISPR‐Cas Systems.” Science Advances 9, no. 12: eadd8911. 10.1126/sciadv.add8911. 36961900 PMC10038342

[omi70014-bib-0051] Saint‐Jean, M. , O. Claisse , C. Le Marrec , and J. Samot . 2025. “Structural and Genetic Diversity of Lysis Modules in Bacteriophages Infecting the Genus *Streptococcus* .” Genes 16, no. 7: 842. 10.3390/genes16070842. 40725499 PMC12294143

[omi70014-bib-0052] Serbanescu, M. A. , M. Cordova , K. Krastel , et al. 2015. “Role of the *Streptococcus mutans* CRISPR‐Cas Systems in Immunity and Cell Physiology.” Journal of Bacteriology 197, no. 4: 749–761. 10.1128/JB.02333-14. 25488301 PMC4334182

[omi70014-bib-0053] Shibata, Y. , Y. Yamashita , and J. R. van der Ploeg . 2009. “The Serotype‐Specific Glucose Side Chain of Rhamnose‐Glucose Polysaccharides Is Essential for Adsorption of Bacteriophage M102 to *Streptococcus mutans* .” Fems Microbiology Letters 294, no. 1: 68–73. 10.1111/j.1574-6968.2009.01546.x. 19493010

[omi70014-bib-0054] Shmakov, S. A. , V. Sitnik , K. S. Makarova , Y. I. Wolf , K. V. Severinov , and E. V. Koonin . 2017. “The CRISPR Spacer Space Is Dominated by Sequences From Species‐Specific Mobilomes.” MBio 8, no. 5: e01397–17. 10.1128/mBio.01397-17. PMC5605939 28928211

[omi70014-bib-0055] Shmakov, S. A. , Y. I. Wolf , E. Savitskaya , K. V. Severinov , and E. V. Koonin . 2020. “Mapping CRISPR Spaceromes Reveals Vast Host‐Specific Viromes of Prokaryotes.” Communications Biology 3, no. 1: 321. 10.1038/s42003-020-1014-1. 32572116 PMC7308287

[omi70014-bib-0056] Sievers, F. , and D. G. Higgins . 2021. “The Clustal Omega Multiple Alignment Package.” Methods in Molecular Biology 2231: 3–16. 10.1007/978-1-0716-1036-7_1. 33289883

[omi70014-bib-0057] Stoler, N. , and A. Nekrutenko . 2021. “Sequencing Error Profiles of Illumina Sequencing Instruments.” NAR Genomics Bioinformatics 3, no. 1: lqab019. 10.1093/nargab/lqab019. 33817639 PMC8002175

[omi70014-bib-0058] Sugai, K. , M. Kawada‐Matsuo , M. Nguyen‐Tra Le , et al. 2023. “Isolation of *Streptococcus mutans* Temperate Bacteriophage With Broad Killing Activity to *S. mutans* Clinical Isolates.” Iscience 26, no. 12: 108465. 10.1016/j.isci.2023.108465. 38089578 PMC10713843

[omi70014-bib-0059] Syed, M. A. , S. Koyanagi , E. Sharma , M. C. Jobin , A. F. Yakunin , and C. M. Levesque . 2011. “The Chromosomal mazEF Locus of *Streptococcus mutans* Encodes a Functional Type II Toxin‐Antitoxin Addiction System.” Journal of Bacteriology 193, no. 5: 1122–1130. 10.1128/JB.01114-10. 21183668 PMC3067577

[omi70014-bib-0060] Tesson, F. , A. Herve , E. Mordret , et al. 2022. “Systematic and Quantitative View of the Antiviral Arsenal of Prokaryotes.” Nature Communications 13, no. 1: 2561. 10.1038/s41467-022-30269-9. PMC9090908 35538097

[omi70014-bib-0061] Tisza, M. J. , and C. B. Buck . 2021. “A Catalog of Tens of Thousands of Viruses From Human Metagenomes Reveals Hidden Associations With Chronic Diseases.” PNAS 118, no. 23: e2023202118. 10.1073/pnas.2023202118. 34083435 PMC8201803

[omi70014-bib-0062] van der Ploeg, J. R. 2007. “Genome Sequence of *Streptococcus mutans* Bacteriophage M102.” Fems Microbiology Letters 275, no. 1: 130–138. 10.1111/j.1574-6968.2007.00873.x. 17711456

[omi70014-bib-0063] van der Ploeg, J. R. 2009. “Analysis of CRISPR in *Streptococcus mutans* Suggests Frequent Occurrence of Acquired Immunity Against Infection by M102‐Like Bacteriophages.” Microbiology 155, no. Pt 6: 1966–1976. 10.1099/mic.0.027508-0. 19383692

[omi70014-bib-0064] Vassallo, C. N. , C. R. Doering , M. L. Littlehale , G. I. C. Teodoro , and M. T. Laub . 2022. “A Functional Selection Reveals Previously Undetected Anti‐Phage Defence Systems in the *E. coli* Pangenome.” Nature Microbiology 7, no. 10: 1568–1579. 10.1038/s41564-022-01219-4. PMC9519451 36123438

[omi70014-bib-0065] Walker, A. R. , and R. C. Shields . 2022. “Investigating CRISPR Spacer Targets and Their Impact on Genomic Diversification of *Streptococcus mutans* .” Frontiers in Genetics 13: 997341. 10.3389/fgene.2022.997341. 36186424 PMC9522601

[omi70014-bib-0066] Wang, J. , W. Dai , J. Li , et al. 2021. “AcrHub: An Integrative Hub for Investigating, Predicting and Mapping Anti‐CRISPR Proteins.” Nucleic Acids Research 49, no. D1: D630–D638. 10.1093/nar/gkaa951. 33137193 PMC7779044

[omi70014-bib-0067] Yi, H. , L. Huang , B. Yang , J. Gomez , H. Zhang , and Y. Yin . 2020. “AcrFinder: Genome Mining Anti‐CRISPR Operons in Prokaryotes and Their Viruses.” Nucleic Acids Research 48, no. W1: W358–W365. 10.1093/nar/gkaa351. 32402073 PMC7319584

[omi70014-bib-0068] Zhao, H. , D. Dufour , J. Zhong , S. G. Gong , P. H. Roy , and C. M. Levesque . 2024. “Decoding Adenine DNA Methylation Effects in *Streptococcus mutans*: Insights into Self‐DNA Protection and Autoaggregation.” Molecular Oral Microbiology 40, no. 2: 82–93. 10.1111/omi.12489. 39624001 PMC11904264

[omi70014-bib-0069] Ziane‐Casenave, S. , O. Claisse , M. Saint‐Marc , M.‐C. Badet , and J. Samot . 2023. Comparison of Different Methods of Identification of Streptococcus mutans Clinical Isolates. SSRN. 10.2139/ssrn.4477726. https://papers.ssrn.com/abstract=4477726.

