# Supplementary figures and images for: Defense Systems and Prophage Detection in Streptococcus mutans Strains
Source: Mol Oral Microbiol. 2025 Nov 11;41(2):57–68. doi: 10.1111/omi.70014 (PMC12964521; doi:10.1111/omi.70014)

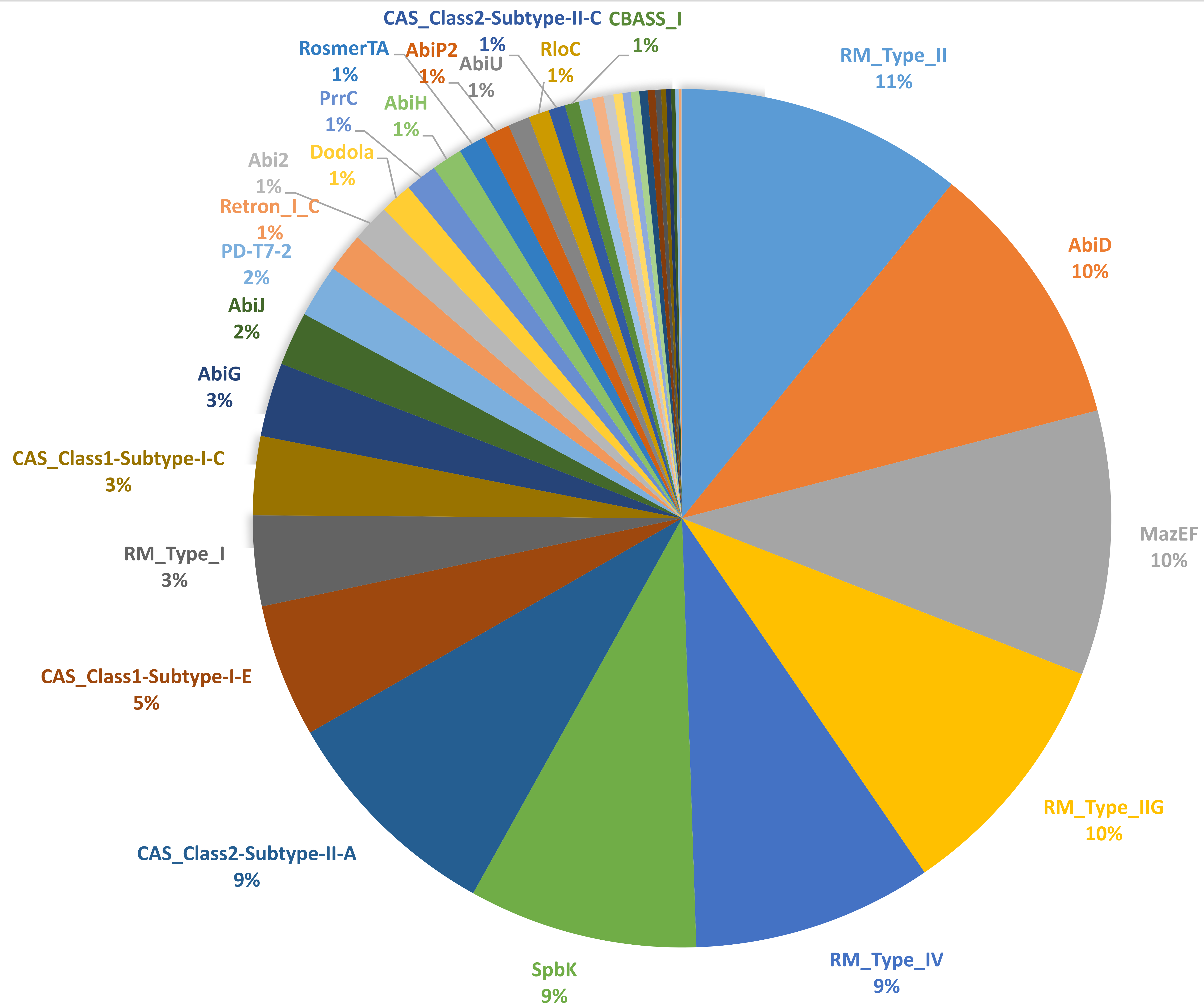

Supplement: Supplementary file 2 — Figure S2(a): Overview of antiphage defense systems (APDSs) identified in the genomes of 478 publicly available S. mutans strains (data retrieved in May 2024). [file OMI-41-57-s003.pdf]

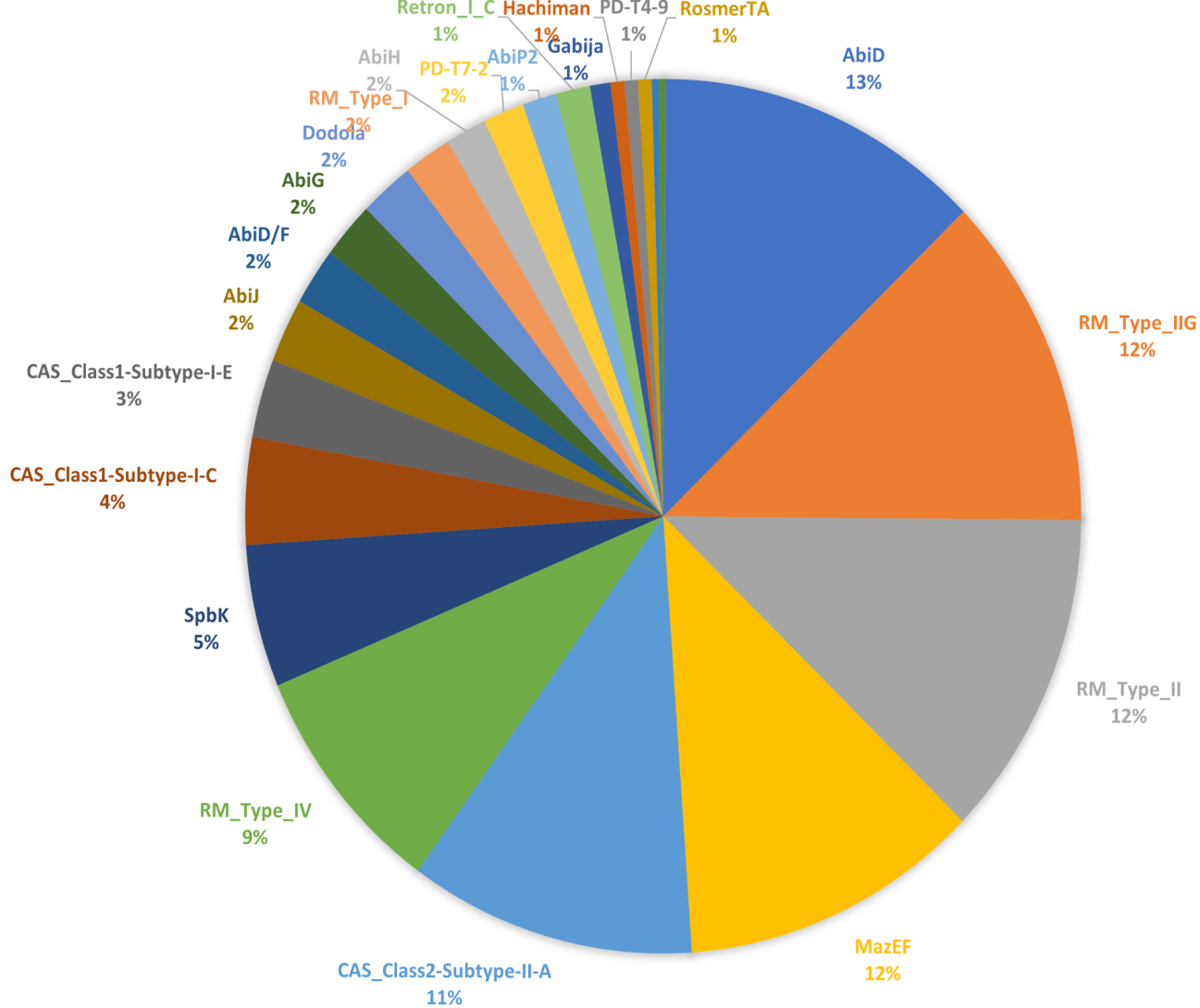

Supplement: Supplementary file 3 — Figure S2(b): APDSs identified in the genomes of 44 newly sequenced S. mutans clinical isolates. [file OMI-41-57-s006.pdf]

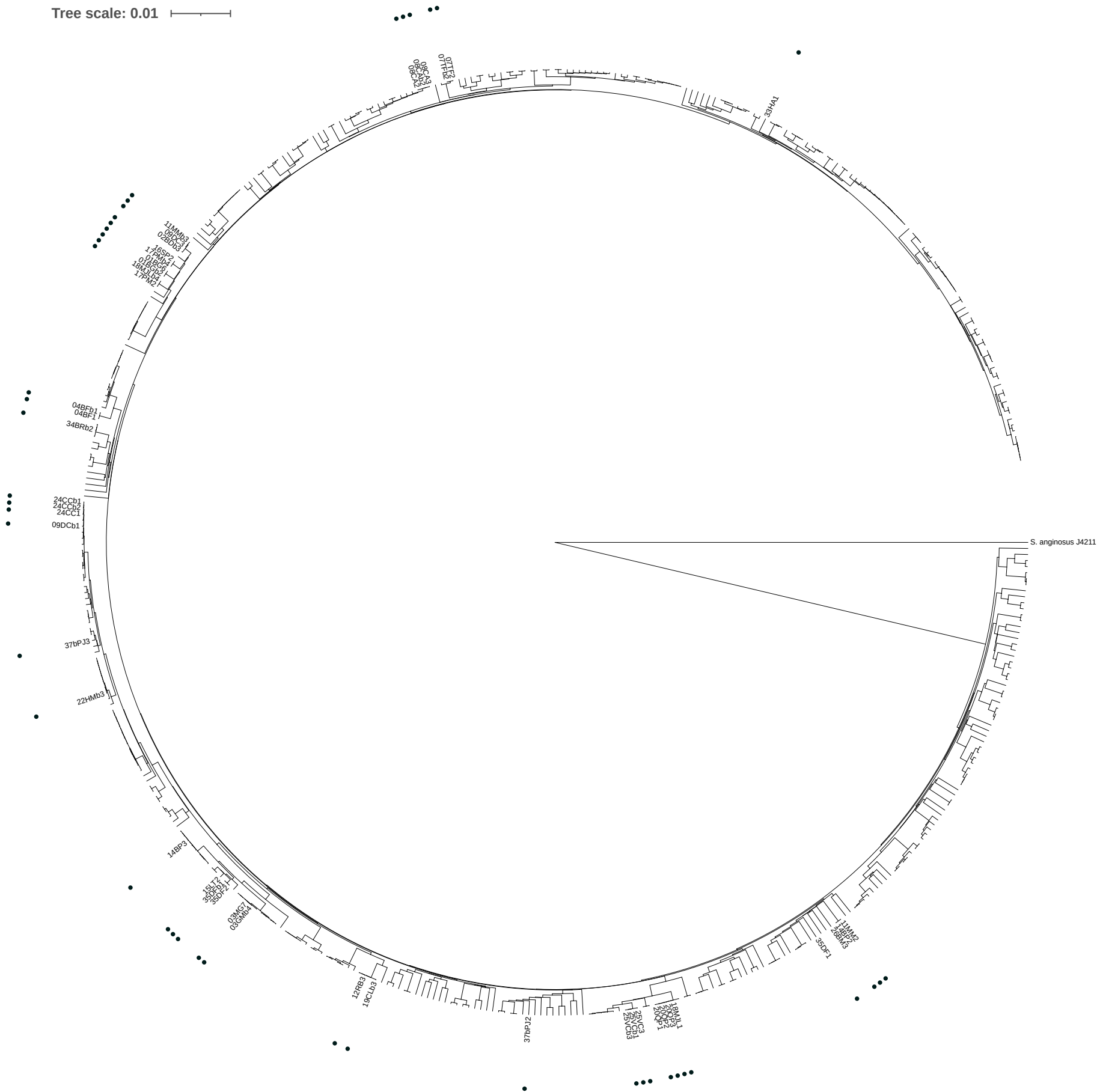

Supplement: Supplementary file 4 — Figure S2(c): Phylogenetic placement of the 44 clinical isolates within the full set of 478 S. mutans genomes. Clinical isolates are marked by black circles and labeled with their strain names. The phylogenetic tree was rooted using Streptococcus anginosus J4211 as an outgroup, given its close yet distinct taxonomic relationship to S. mutans. [file OMI-41-57-s005.pdf]
