# Supplementary material for: Defense Systems and Prophage Detection in Streptococcus mutans Strains
Source: Mol Oral Microbiol. 2025 Nov 11;41(2):57–68. doi: 10.1111/omi.70014 (PMC12964521; doi:10.1111/omi.70014)
Supplement: Supplementary file 5 — Table S3: CRISPR‐Cas systems and their associated direct repeats in the 44 clinical isolates of S. mutans from this study. [file OMI-41-57-s004.pdf]

Strain	CAS_Type (spacer count)	Direct repeats			phage	Acr
01BG6	II-A (8)	gtttttgtactctcaagatttaagtaactgtacaac				
	I-C (22)	gtcgcacccttcacgggtgcgtggattgaaat				
01BGb2	II-A (8)	gtttttgtactctcaagatttaagtaactgtacaac				
	I-C (22)	gtcgcacccttcacgggtgcgtggattgaaat				
02BDb3	II-A (15)	gttttagagc**tg**tgttgttt**tcgaatgg**t**t**c**c**aaaa**c**	gttttagagc**ca**tgttagtt**actgatt**tac**t**aaaa**t**			
	I-C (2)	**cc**gtcgcacccttcacgggtgcgtggattgaaata	**ac**gtcgcacccttcacgggtgcgtggattgaaata	**ag**gtcgcacccttcacgggtgcgtggattgaaat**t**		
03GMb4	II-A (0)					
03MG7	II-A (0)					
04BF1	II-A (20)	gttttagagctgtgttgtttcgaatggttccaaaac	gttttagagctgtgttgtttcgaatggttccaaaat	gttttagagccatgttagttactgatttactaaaac		
04BFb1	II-A (20)	gttttagagctgtgttgtttcgaatggttccaaaac	gttttagagctgtgttgtttcgaatggttccaaaat	gttttagagccatgttagttactgatttactaaaac		
07TF2	II-A (29)	gttttagagctgtgttgtttcgaatggttccaaaac				
	I-C (11)	gtcgcacccttcacgggtgcgtggattgaaat				
	I-E (26)	attttacccgcacgagcgggggtgatcc				
07TFb2	II-A (26)	gttttagagctgtgttgtttcgaatggttccaaaac	gttttagagctatgttgtttcgaatggttccaaaac			
	I-C (11)	gtcgcacccttcacgggtgcgtggattgaaat				
	I-E (24)	attttacccgcacgagcgggggtgatcc				
08CA2	II-A (5)	gtttttgtactctcaagatttaagtaactgtacaac				
	I-C (12)	gtcgcacccttcacgggtgcgtggattgaaat	gtcgcaccctttaaaggttgggtttgcttttt			
08CA3	II-A (5)	gtttttgtactctcaagatttaagtaactgtacaac				
	I-C (12)	gtcgcacccttcacgggtgcgtggattgaaat	gtcgcaccctttaaaggttgggtttgcttttt			
08CAb2	II-A (5)	gtttttgtactctcaagatttaagtaactgtacaac				
	I-C (12)	gtcgcacccttcacgggtgcgtggattgaaat	gtcgcaccctttaaaggttgggtttgcttttt			
09DC3	II-A (15)	gttttagagctgtgttgtttcgaatggttccaaaac				
	I-C (9)	gtcgcacccttcacgggtgcgtggattgaaat				
09DCb1	II-A (30)	gttttagagctgtgttgtttcgaatggttccaaaac				
11MM2	II-A (12)	gttttagagctgtgttgtttcgaatggttccaaaac	gttttagagccatgttagttactgatttactaaaac			
11MMb3	II-A (15)	gttttagagctgtgttgtttcgaatggttccaaaac	gttttggaaccattcgaaacaacacagctctaaaac			
	I-C (9)	gtcgcacccttcacgggtgcgtggattgaaat				
12RB3	II-A (7)	gttttagagctgtgttgtttcgaatggttccaaaac	gttttagagccatgttagttactgatttactaaaat			
14BP2	II-A (12)	gttttagagctgtgttgtttcgaatggttccaaaac	gttttagagccatgttagttactgatttactaaaac			

14BP3	II-A (2)	gttttagagctgtgttgtttcgaatggttccaaaac	gttttagagctgtgttgtttcgaatggttccaaaat			
	I-E (16)	attttacccgcacgagcgggggtgatcc				
15LT2	II-A (35)	gttttagagctgtgttgtttcgaatggttccaaaac	gttttagagctgtgttgtttcgaatggttccaaaat	gttttagagccatgttagttactgatttactaaaac		
	I-E (19)	attttacccgcacgagcgggggtgatcc				
16SP2	II-A (10)	gttgtacagttacttaaatcttgagagtacaaaaac	gttgtacagttacttaaatcttgagagtacaaaaaa	ggatcacccccgctcgtgcgggtaaaat		
	I-C (15)	gtcgcacccttcacgggtgcgtggattgaaat	gtcgcacccttcacgggtgcgtgggttgaaat			
	I-E (76)	attttacccgcacgagcgggggtgatcc				
17PM2	II-A (29)	gtttttgtactctcaagatttaagtaactgtacaac				
	I-C (27)	gtcgcacccttcacgggtgcgtggattgaaat	gtcgcaccctttaaaggttgggtttgtttttt			
	I-E (15)	attttacccgcacgagcgggggtgatcc	attttactcgcacgagcgggggtgatcc			
17PMb4	II-A (10)	gtttttgtactctcaagatttaagtaactgtacaac	ttttttgtactctcaagatttaagtaactgtacaac			
	I-C (15)	gtcgcacccttcacgggtgcgtggattgaaat	gtcgcacccttcacgggtgcgtgggttgaaat			
	I-E (83)	attttacccgcacgagcgggggtgatcc				
18MJL1	II-A (8)	gttttagtaaatcagtaactaacatggctctaaaac	attttggaaccattcgaaacaacacagctctaaaac	gttttggaaccattcgaaacaacacagctctaaaac		
18MJLb4	II-A (29)	gtttttgtactctcaagatttaagtaactgtacaac				
	I-C (27)	gtcgcacccttcacgggtgcgtggattgaaat	gtcgcaccctttaaaggttgggtttgtttttt			
	I-E (15)	attttacccgcacgagcgggggtgatcc	attttactcgcacgagcgggggtgatcc			
19CLb3	II-A (8)	gttttagagctgtgttgtttcgaatggttccaaaac	gttttagagccatgttagttactgatttactaaaat			
20QP1	II-A (7)	gttttagtaaatcagtaactaacatggctctaaaac	attttggaaccattcgaaacaacacagctctaaaac	gttttggaaccattcgaaacaacacagctctaaaac		
20QP2	II-A (7)	gttttagtaaatcagtaactaacatggctctaaaac	attttggaaccattcgaaacaacacagctctaaaac	gttttggaaccattcgaaacaacacagctctaaaac		
20QP3	II-A (7)	gttttagagctgtgttgtttcgaatggttccaaaac	gttttagagctgtgttgtttcgaatggttccaaaat	gttttagagccatgttagttactgatttactaaaac		
22HMb3	II-A (35)	gttttagagctgtgttgtttcgaatggttccaaaac	gttttagagccatgttagttactgatttactaaaac			
24CC1	II-A (18)	gttttagagctgtgttgtttcgaatggttccaaaac	gttttagagccatgttagttactgatttactaaaac			
24CCb1	II-A (23)	gttttagagctgtgttgtttcgaatggttccaaaac	gttttagagccatgttagttactgatttactaaaac			
24CCb2	II-A (24)	gttttagagctgtgttgtttcgaatggttccaaaac	gttttagagccatgttagttactgatttactaaaac			
25VC3	II-A (6)	attttggaaccattcgaaacaacacagctctaaaac	gttttggaaccattcgaaacaacacagctctaaaac			
25VCb1	II-A (6)	gttttagagctgtgttgtttcgaatggttccaaaac	gttttagagctgtgttgtttcgaatggttccaaaat			
25VCb3	II-A (6)	attttggaaccattcgaaacaacacagctctaaaac	gttttggaaccattcgaaacaacacagctctaaaac			
26BM3	II-A (11)	gttttagagctgtgttgtttcgaatggttccaaaac	gttttagagccatgttagttactgatttactaaaat			
34BRb2	II-A (5)	ggttttagagctgtgttgtttcgaatggttccaaaac	cgttttagagctgtgttgtttcgaatggttccaaaac	gttttggaaccattcgaaacaacacagctctaaaac		
35DF1	I-C (2)	gtcgcacccttcacgggtgcgtggattgaaatt	gtcgcacccttcacgggtgcgtggattgaaata	gtcgcaccctttaaaggttgggtttgcttttta		
35DF2	II-A (8)	gttttagagctgtgttgtttcgaatggttccaaaac	gttttagagctgtgttgtttcgaatggttccaaaat	gttttagagccatgttagttactgatttactaaaac		
	I-E (7)	attttacccgcacgagcgggggtgatccc	attttacccgcacgagcgggggtgatcct			

35DFb1	II-A (8)	gttttagagctgtgttgtttcgaatggttccaaaac	gttttagagctgtgttgtttcgaatggttccaaaat	gttttagagccatgttagttactgatttactaaaac		
	I-E (7)	attttacccgcacgagcgggggtgatccc	attttacccgcacgagcgggggtgatcct			
37bPJ2	II-A (6)	gttttagagctgtgttgtttcgaatggttccaaaac	gttttagagccatgttagttactgatttactaaaat		yes	AcrIIA5
37bPJ3	II-A (12)	gttttagagctgtgttgtttcgaatggttccaaaac	gttttagagctgtgttgtttcgaatggttccaaagc	gttttagagccatgttagttactgatttactaaaac		
	I-A (0)					
	I-E (11)	attttacccgcacgagcgggggtgatcc	attttacccgcacgagcggaggtgatcc			
33HA1	nd					
