# Supplementary material for: Defense Systems and Prophage Detection in Streptococcus mutans Strains
Source: Mol Oral Microbiol. 2025 Nov 11;41(2):57–68. doi: 10.1111/omi.70014 (PMC12964521; doi:10.1111/omi.70014)
Supplement: Supplementary file 6 — Table S4: Overview of BLAST matches for 117 spacers out of 579 unique sequences: associated bacterial strains and phages. [file OMI-41-57-s001.pdf]

product	strain/phage
AAA domain protein	isolate ctNo011
AAA family ATPase	phiKSM96
anti-CRISPR protein	phiKSM96
ATP-binding protein	phiKSM96
bifunctional DnaQ family exonuclease/ATP-dependent helicase	LAB761
capsid and scaffold protein	smHBZ8
capsid protein	APCM01
class I SAM-dependent methyltransferase	phiKSM96
Clp protease	phiKSM96
conjugal transfer protein	NCH105
DEAD/DEAH box helicase	KCOM 1054
DEAD/DEAH box helicase	KCOM 1054
distal tail protein	ctNo011
DNA cytosine methyltransferase	phiKSM96
DnaC-like helicase loader	M102AD
endolysin	M102
formate C-acetyltransferase	LAB761
glutamate synthase large subunit	LAB761
head-tail connector protein	phiKSM96
holin	APCM01
hypothetical protein	KCOM 1054
hypothetical protein	phiKSM96
hypothetical protein	OMZ175
hypothetical protein	KCOM 1054
hypothetical protein	phage smHBZ8
hypothetical protein	phiKSM96
hypothetical protein	COCC33-14R
hypothetical protein	Javan237
hypothetical protein	phiKSM96
hypothetical protein	M102AD
hypothetical protein	OMZ175
hypothetical protein	KCOM 1054
hypothetical protein	KCOM 1054
hypothetical protein	phiKSM96
hypothetical protein"	UA159
ImmA/IrrE family metallo-endopeptidase	KCOM 1054
intergenic	LP13
intergenic	GS-5
intergenic	NCTC10832
intergenic	smHBZ8
intergenic	APCM01
intergenic	MD
intergenic	NCTC10832
intergenic	phiKSM96
intergenic	NCTC10832
intergenic	NCTC10832
intergenic	NCTC10832
intergenic	NCTC10832

intergenic	NCTC10832
intergenic	GS-5
intergenic	M102
intergenic	M102
intergenic	M102
lantibiotic ABC transporter permease	LAB761
lysin	smHBZ8
lysin	phiKSM96
minor tail protein	phage M102
minor tail protein	phage M102
minor tail protein	M102
minor tail protein	M102AD
minor tail protein	M102
minor tail protein	M102AD
minor tail protein	M102
minor tail protein	APCM01
minor tail protein	M102AD
phage tail protein	KCOM 1054
phage tail protein	KCOM 1054
phage tail protein	KCOM 1054
phage tail protein	KCOM 1054
phage tail protein	KCOM 1054
phage tail protein	KCOM 1054
portal protein	phiKSM96
portal protein	phiKSM96
portal protein	phiKSM96
predicted ATPases	LP13
Protein of unknown function (DUF669)	ctNo011
putative endolysin	phage M102
putative major tail protein	M102
putative minor structural protein	M102
putative phage capsid protein	phage M102
putative portal protein	M102AD
putative tape measure protein	M102AD
recombinase family protein	phiKSM96
Regulatory protein repA	ctHbp13
ribonuclease Y	LAB761
single strand DNA binding protein	M102
single-stranded DNA-binding protein	smHBZ8
site-specific DNA-methyltransferase	strain S1
site-specific DNA-methyltransferase	strain S1
spacer	strain MD
spacer	strain MD
spacer	FDAARGOS 1458
spacer	LP13
spacer	LP13
spacer	FDAARGOS 1458
spacer	FDAARGOS 1458
spacer	NN2025
spacer	DPC6143

spacer	DPC6143
STRUCTURAL MAINTENANCE OF CHROMOSOMES PROTEIN	ctNo011
sugar ABC transporter permease	LAB761
tail family protein	phiKSM96
tail length tape-measure protein	smHBZ8
tail length tape-measure protein	smHBZ8
tail protein	phiKSM96
tail protein	ctNo011
tail protein	M102
tape measure protein	phiKSM96
tape measure protein	phiKSM96
tape measure protein	phiKSM96
terminase large subunit	M102AD
terminase large subunit	smHBZ8
terminase small subunit	M102AD
terminase small subunit	M102AD
Transporter	LP13
transposon protein	UA159
transposon protein	UA159
