# Supplementary material for: Defense Systems and Prophage Detection in Streptococcus mutans Strains
Source: Mol Oral Microbiol. 2025 Nov 11;41(2):57–68. doi: 10.1111/omi.70014 (PMC12964521; doi:10.1111/omi.70014)
Supplement: Supplementary file 7 — Figure S5: Alignment of the AcrIIA5 protein from prophage phi_37bPJ2 with AcrIIA5 proteins from other phages infecting Streptococcus mutans. [file OMI-41-57-s002.pdf]

```
                         1                                                                    70
AcrIIA5_phage_37bPJ2     MAFGTRRYNS YRKRSFNRSD KQRREYAQAM EELEQTFENL EDWNLSSMKD SAYKDYDKYE VRLSNHSADN
AcrIIA5_phage_NLML9-1    MAFGKRRYNS YRKRSFNRSD KQRREYAQAM EELEQTFENL EGWNLSSMKD SAYKDYDKYE VRLSNHSADN
AcrIIA5_phage_KSM96      MAFGKRRYNS YRKRSFNRSD KQRREYAQAM EELEQTFENL EDWNLSSMKD SAYKDYDKYE VRLSNHSADN
Consensus                MAFGkRRYNS YRKRSFNRSD KQRREYAQAM EELEQTFENL EdWNLSSMKD SAYKDYDKYE VRLSNHSADN

                         71                                                                  140
AcrIIA5_phage_37bPJ2     QYHNLQDGKL IINIKASKMN FVWIIENKLD AILEKVNKLD LSKYRFINAT SLDHDIKCYY KNYKTKKDVI
AcrIIA5_phage_NLML9-1    QYHNLQDGKL IINIKASKMN FVWIIENKLD AILEKVNKLD LSKYRFINAT SLDHDIKCYY KNYKTKKDVI
AcrIIA5_phage_KSM96      QYHNLQYGKL IINIKASKMN FVWIIENKLD AILEKVNKLD LSKYRFINAT SLDHDIKCYY KNYKTKKDVI
Consensus                QYHNLQdGKL IINIKASKMN FVWIIENKLD AILEKVNKLD LSKYRFINAT SLDHDIKCYY KNYKTKKDVI
```
